# Multiparticle collision dynamics simulations of a squirmer in a nematic fluid

**DOI:** 10.1140/epje/s10189-021-00072-3

**Published:** 2021-05-03

**Authors:** Shubhadeep Mandal, Marco G. Mazza

**Affiliations:** 1 Department of Mechanical Engineering, Indian Institute of Technology Guwahati, Guwahati, Assam 781039 India; 2 Max-Planck-Institute for Dynamics and Self-Organization, Am Fassberg 17, 37077 Göttingen, Germany; 3 Interdisciplinary Centre for Mathematical Modelling and Department of Mathematical Sciences, Loughborough University, Leicestershire LE11 3TU, Loughborough, United Kingdom

## Abstract

**Abstract:**

We study the dynamics of a squirmer in a nematic liquid crystal using the multiparticle collision dynamics (MPCD) method. A recently developed nematic MPCD method [Phys. Rev. E 99, 063319 (2019)] which employs a tensor order parameter to describe the spatial and temporal variations of the nematic order is used to simulate the suspending anisotropic fluid. Considering both nematodynamic effects (anisotropic viscosity and elasticity) and thermal fluctuations, in the present study, we couple the nematic MPCD algorithm with a molecular dynamics (MD) scheme for the squirmer. A unique feature of the proposed method is that the nematic order, the fluid, and the squirmer are all represented in a particle-based framework. To test the applicability of this nematic MPCD-MD method, we simulate the dynamics of a spherical squirmer with homeotropic surface anchoring conditions in a bulk domain. The importance of anisotropic viscosity and elasticity on the squirmer’s speed and orientation is studied for different values of self-propulsion strength and squirmer type (pusher, puller or neutral). In sharp contrast to Newtonian fluids, the speed of the squirmer in a nematic fluid depends on the squirmer type. Interestingly, the speed of a strong pusher is smaller in the nematic fluid than for the Newtonian case. The orientational dynamics of the squirmer in the nematic fluid also shows a non-trivial dependence on the squirmer type. Our results compare well with existing experimental and numerical data. The full particle-based framework could be easily extended to model the dynamics of multiple squirmers in anisotropic fluids.

**Graphic abstract:**

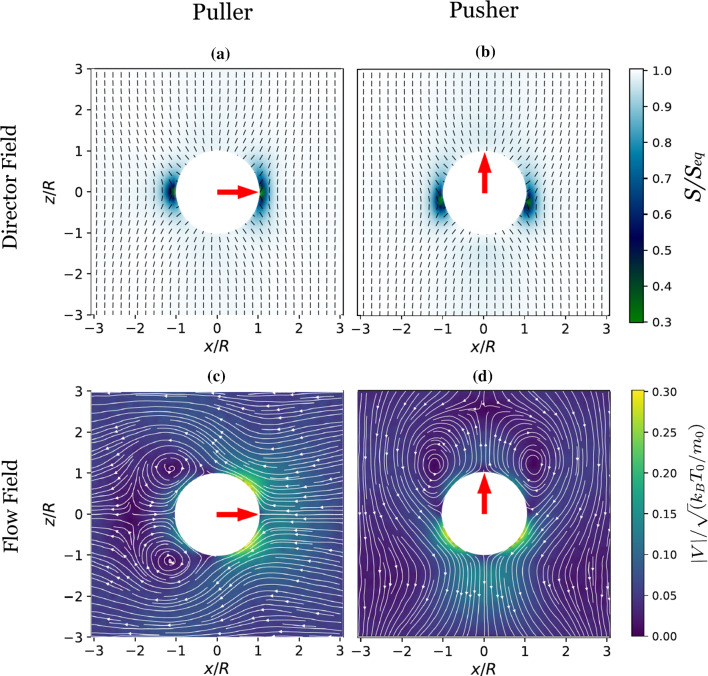

## Introduction

Microswimmers are natural or artificial self-driven entities which are capable of converting stored or ambient energy into a systematic motion in a suspending fluid medium [1]. Recently, artificial microswimmers have fascinated several researchers because of their futuristic applications in drug delivery, disease monitoring, minimally invasive surgery, swarm robotics, etc. [2–5]. In order to achieve these technological goals, a major challenge is to gain control over trajectory and orientation of the microswimmers in complex environments. These complex environments not only include geometric complexities, but also the complexity in the rheology of suspending fluids [6]. Here we are interested in a rheologically complex fluid, namely nematic liquid crystals (LCs), having anisotropic viscosity and elasticity [7].

While the dynamics of microswimmers in Newtonian fluids are reasonably well studied, the dynamics of microswimmers in anisotropic and elastic environments such as LCs were given due consideration only recently [7–9]. Unlike Newtonian fluids, LCs possess long-range orientational order due to their asymmetric molecular structure. This orientational order gives rise to anisotropic viscosity and elasticity of the medium which dramatically alters the behaviour of microswimmers in an LC medium as compared to a Newtonian medium. Instead of performing random trajectories in three-dimensional geometry, recent experiments have shown that flagellated microorganisms (e.g. *E. coli*, *B. subtilis*, and *P. mirabilis*) move parallel to the nematic director (anisotropy axis) in a biocompatible LC, namely a solution of water and biocompatible compound DSCG (disodium cromoglycate) [10–15]. The motion of elongated bacteria parallel to the nematic director can be explained by the minimization of elastic energy of the medium around a rodlike body. The orientation of these elongated bacteria away from the nematic director is always resisted by an elastic torque. Recently, Lintuvuori et al. [16] have studied the reorientation dynamics of a spherical squirmer in nematic LC and found that at steady state pushers (pullers) swim parallel (perpendicular) to the nematic director. The analytical calculation showed that the reorientation dynamics of a spherical squirmer is governed by a nematodynamic toque associate with the squirmer’s flow field and anisotropic viscosity of the suspending medium [16–18]. This unique behaviour of the microswimmers was utilized to transport cargo of cells/particles along prescribed pathways determined by the local nematic director field over long distances [14, 19, 20]. More recently, the dynamics of bacteria in the presence of bounding walls [19, 21] and defects [8, 22–26] have been investigated.

A closer look into the theoretical literature shows that dynamics of microswimmers in LCs have been investigated using either the lattice Boltzmann method [16] or finite element method [18]. These methods solve the nematodynamics but do not include thermal fluctuations. It is well established that thermal fluctuations play an important role in governing the dynamics of microswimmers in Newtonian fluids. Thus, it would be quite useful to have a simulation model that can be used to study microswimmers in combined presence of nematodynamic effects and thermal fluctuations. Towards this, the multiparticle collision dynamics (MPCD) seems quite promising. MPCD, originally proposed by Malevanets and Kapral as stochastic rotation dynamics (SRD) [27], is a mesoscopic particle-based simulation method for fluids [28, 29]. Alternating streaming and collision steps are performed in such a way that mass, momentum and energy (or temperature) are conserved locally so that at long time and large length-scale the Navier-Stokes hydrodynamics with thermal fluctuations is recovered. The particle nature of the MPCD method makes it quite successful in solving several nonequilibrium soft matter systems ranging from colloids [30], viscoelastic fluids and polymers [31, 32], liquid crystals [33, 34], binary fluids [35], biological cells and vesicles [36], and microswimmers [37, 38]. Recently, we have extended the multiparticle collision dynamics (MPCD) method for nematic LCs by combining anisotropic viscosity, elasticity and thermal fluctuations [39]. This nematic MPCD model which incorporates the tensorial nematic orinetational order is more general than other fluctuating nematodynamics models [33, 34]. In the present study, we build on our model and incorporate a moving squirmer in nematic LCs. Our goal is to produce a full particle-based simulation technique for microswimmers in nematic LCs.

## Model

In the following we provide a detail description of the nematic MPCD-MD algorithm which we use to study the dynamics of a squirmer moving in a nematic LC medium.

### Modelling of nematic LC: nematic MPCD algorithm

#### System description

**Fig. 1:**
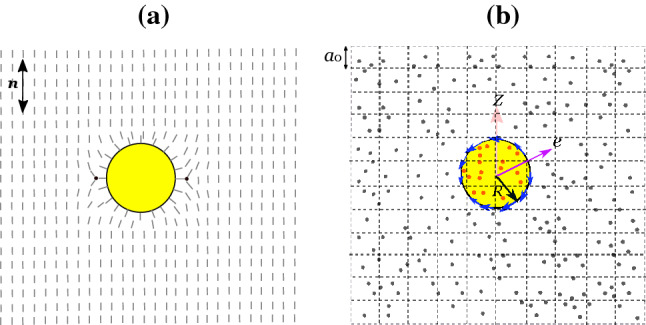
**a** Schematic representation of the distribution of nematic director field around a spherical particle in unbounded domain. Homeotropic anchoring leads to the formation of a Saturn ring around the spherical inclusion. **b** Schematic representation of a squirmer (with radius *R* and orientation ${\varvec{e}}$) in a fluid. The fluid particles are presented by black dots, while the virtual particles are represented by red dots. The bulk director field, ${\varvec{n}}$, is oriented along the *z*-axis

The basic MPCD method employs pointlike particles to represent the fluid. These particles are not fluid molecules rather they represent a parcel of fluid. To describe a Newtonian fluid on a coarse-grained level, the fluid is represented by $N_{f}$ pointlike particles with mass $m_{0}$, position ${\varvec{r}}_{i}$ and velocity ${\varvec{v}}_{i}$. In sharp contract to Newtonian fluids, nematic LCs are made of asymmetric (e.g. rodlike) molecules which produce long-range orientational order. A complete description of this orientational order can be given by a tensor order parameter. To incorporate the orientational order in the present particle-based framework, we assign a tensor order parameter ${\varvec{q}}_{i}$ (symmetric and traceless 2$^\text {nd}$ order tensor) to each MPCD particle in addition to position and velocity [39]. In the nematic MPCD framework, in addition to particle-level quantities, we also have cell-level quantities. The simulation domain is divided into cubic cells of side length $a_{0}$ (see Fig. 1). The particle-level quantities are related to the cell-level quantities in the following way: cell-level velocity field ${\varvec{V}} = \frac{1}{N_{c}} \sum _{j \in \text {cell}} {\varvec{v}}_{j}$, and cell-level tensor order parameter ${\varvec{Q}} = \frac{1}{N_{c}} \sum _{j \in \text {cell}} {\varvec{q}}_{j}$, where $N_{c}$ is the number of particles in the given cell under consideration.

#### Evolution of tensor order parameter

The evolution of the particle-based tensor order parameter ${\varvec{q}}_{i}$ is given by [39]

1 $$\begin{aligned} {\varvec{q}}_{i}(t + \Delta t) = {\varvec{q}}_{i}(t) + {\varvec{g}}_{i}(t) \Delta t, \end{aligned}$$

where ${\varvec{g}}_{i}$ is a cell-level tensor quantity. The components of ${\varvec{g}}$ are given as

2 $$\begin{aligned} g_{\alpha \beta } = g_{\alpha \beta }^{\text {mol}} + g_{\alpha \beta }^{\text {vel--ori}} + g_{\alpha \beta }^{\text {Lag}}, \end{aligned}$$

where $g_{\alpha \beta }^{\text {mol}}$ represents the molecular field which gives rise to the nematic-isotropic phase transition, $g_{\alpha \beta }^{\text {vel--ori}}$ represents the velocity-orientation coupling, and $g_{\alpha \beta }^{\text {Lag}}$ represents the enforcement of tracelessness and symmetry of ${\varvec{q}}_{i}$ using Lagrange multipliers [40]. The molecular field is given by $g_{\alpha \beta }^{\text {mol}} = \frac{1}{\mu _{1}} H_{\alpha \beta }$, where $\mu _{1}$ is viscosity coefficient and $H_{\alpha \beta }$ is given by the Landau–de Gennes theory as [41] $H_{\alpha \beta } = L \partial _{\mu }^{2} Q_{\alpha \beta } - \alpha _{F} Q_{\alpha \beta } + 3\beta _{F} Q_{\alpha \mu }Q_{\beta \mu } - 4\gamma _{F} Q_{\alpha \beta }Q_{\mu \nu }Q_{\mu \nu }$, where *L* is elastic constant (note that we have assumed the one-elastic-constant approximation) and $\alpha _{F}$, $\beta _{F}$ and $\gamma _{F}$ are three phenomenological material constants. The velocity-orientation coupling is given by $g_{\alpha \beta }^{\text {vel--ori}} = - \frac{\mu _{2}}{2 \mu _{1}} A_{\alpha \beta } + (Q_{\alpha \mu }W_{\mu \beta } - W_{\alpha \mu }Q_{\mu \beta })$, where $\mu _{2}$ is viscosity coefficient, $A_{\alpha \beta } = \frac{1}{2}(\partial _{\alpha }V_{\beta } + \partial _{\beta }V_{\alpha })$ is the symmetric part of velocity gradient tensor and $W_{\alpha \beta } = \frac{1}{2}(\partial _{\alpha }V_{\beta } - \partial _{\beta }V_{\alpha })$ is the anti-symmetric part of the velocity gradient tensor. The traceless and symmetry of ${\varvec{q}}$ is imposed using $g_{\alpha \beta }^{\text {Lag}} = \frac{1}{\mu _{1}}(\lambda \delta _{\alpha \beta } + \lambda _{\mu } \epsilon _{\mu \alpha \beta })$, where $\lambda$ and $\lambda _{\mu }$ are Lagrange multipliers. A central difference discretization scheme is used to calculate $g_{\alpha \beta }$ for each cell.

#### Streaming step

In the basic MPCD method, streaming and collision steps are performed in such a way that the mass, linear momentum, angular momentum and temperature of the fluid remain constant. This scheme effectively combines the hydrodynamics (the Navier–Stokes behavior at a coarse-grained level) and thermal fluctuations. For Newtonian fluids, particles are moved ballistically in the streaming step over a time interval $\Delta t$ (referred to as collision time). However, for nematic LCs the streaming step is modified by incorporating the effects of anisotropic viscous stress and elastic stress in the following form [39]

3 $$\begin{aligned}&{\varvec{r}}_{i}(t+\Delta t) = {\varvec{r}}_{i}(t) + {\varvec{v}}_{i}(t) \Delta t + {\varvec{f}}_{i}(t) \frac{\Delta t^{2}}{2m_{0}}, \end{aligned}$$

4 $$\begin{aligned}&{\varvec{v}}_{i}(t+\Delta t) = {\varvec{v}}_{i}(t) + {\varvec{f}}_{i}(t) \frac{\Delta t}{m_{0}}, \end{aligned}$$

where ${\varvec{f}}_{i}$ is a cell-level force. The components of ${\varvec{f}}$ are $f_{\beta } = \frac{a_{0}^{3}}{N_{c}} \partial _{\alpha }(\sigma _{\alpha \beta }^{v,\text {aniso}} + \sigma _{\alpha \beta }^{e})$, where $\sigma _{\alpha \beta }^{v,\text {aniso}}$ is the anisotropic viscous stress and $\sigma _{\alpha \beta }^{e}$ is the elastic stress of the form [40]

5 $$\begin{aligned} \begin{aligned} \sigma _{\alpha \beta }^{v,\text {aniso}} =&\, \beta _{1}Q_{\alpha \beta }Q_{\mu \nu }A_{\mu \nu } + \beta _{5}Q_{\alpha \mu }A_{\mu \beta } + \beta _{6}Q_{\beta \mu }A_{\mu \alpha } \\&+ \frac{1}{2} \mu _{2}N_{\alpha \beta } - \mu _{1}Q_{\alpha \mu }N_{\mu \beta } + \mu _{1}Q_{\beta \mu }N_{\mu \alpha }, \end{aligned} \end{aligned}$$

6 $$\begin{aligned} \sigma _{\alpha \beta }^{e} = - L \partial _{\alpha }Q_{\mu \nu }\partial _{\beta }Q_{\mu \nu },\nonumber \\ \end{aligned}$$

where $\beta _{1}$, $\beta _{5}$ and $\beta _{6}$ are viscosity coefficients, and $N_{\alpha \beta } = D_{t}Q_{\alpha \beta } + W_{\alpha \mu }Q_{\mu \beta } - Q_{\alpha \mu }W_{\mu \beta }$ represents the corotational derivative with $D_{t} \equiv \partial _{t} + V_{\mu }\partial _{\mu }$ as the material time derivative. Note that the central idea behind this modified streaming step is to first calculate the force on each cell and then distribute that force among the particles present in that cell [39]. To calculate the force, we need to take divergence of the anisotropic viscous stress and elastic stress. This cell-level divergence can be calculated by employing a central difference discretization scheme. This scheme ensures that the total force on all the particles vanishes and does not lead to any macroscopic drift in momentum in equilibrium condition. Similar implementation in MPCD in the context of binary fluid mixtures can be found in [35].

#### Collision step

To perform the collision step at fixed discrete time intervals, the simulation domain is divided into small cubic cells of length $a_{0}$. The particles are sorted into collision cells and an instantaneous momentum exchange is performed among all the particles in a cell. To perform the collision step we choose the MPC-AT+a collision rule which conserves linear momentum and angular momentum locally in each cell [42, 43]. In this collision rule the relative velocities of particles are drawn from a Gaussian distribution (Andersen-thermostat) with zero mean and standard deviation $\sqrt{k_{B}T_{0}/m_{0}}$ so that the cell-wise temperature is maintained at constant value $T_{0}$ even in nonequilibrium condition (e.g. presence of external force or flow). Thus, the collision step effectively gives rise to the isotropic viscous stress in an isothermal condition. The velocity after collision step is given by

7 $$\begin{aligned} {{\varvec{v}}}_{i} (t + \Delta t)&= \frac{1}{N_{c}} \sum _{j \in \text {cell}} {\varvec{v}}_{j} (t) + {{\varvec{v}}}_{i}^{\mathrm{{ran}}} - \frac{1}{N_{c}}\sum _{j \in \text {cell}}{{\varvec{v}}}_{j}^{\mathrm{{ran}}} \nonumber \\&+ {{\varvec{\Pi }}_{c}^{-1} \sum _{j \in \text {cell}}[{\varvec{r}}_{j,c} \times ({\varvec{v}}_{j} - {\varvec{v}}^{\mathrm{{ran}}}_{j})] \times {\varvec{r}}_{i,c}},\nonumber \\ \end{aligned}$$

where $N_{c}$ is the particle (number) density in the cell, ${\varvec{v}}_{i}^{\mathrm{{ran}}}$ is the fluctuating part of velocity, ${\varvec{\Pi }}_{c}$ is the moment-of-inertia tensor of the particles in a reference frame at center-of-mass of the cell, ${\varvec{r}}_{j,c} = {\varvec{r}}_{j} - {\varvec{r}}_{c}$ is the relative position of particle *j* in the cell relative to the center-of-mass of the cell, and ${\varvec{r}}_{c}$ is the center-of-mass of the cell. The Cartesian velocity components of ${\varvec{v}}_{i}^{\mathrm{{ran}}}$ are drawn from a Gaussian distribution with zero mean and standard deviation $\sqrt{k_B T_0/m_0}$. The Galilean invariance is violated due to the partitioning of the system into cells. The Galilean invariance is re-established by randomly shifting the grid before performing collision step [44].

### Modelling of the squirmer in nematic LC: nematic MPCD-MD algorithm

The particle-based framework of MPCD makes it straightforward to model the dynamics of embedded particles (e.g. colloids, swimmers, etc.) in a fluid medium by coupling the MPCD algorithm with molecular dynamics (MD) like scheme for embedded particles [38].

#### Squirmer model

The squirmer is a rigid spherical particle of radius *R* with prescribed surface velocity [45] ${\varvec{v}}_{s} = B_{1} \sin \theta (1 + \beta \sin \theta \cos \theta ) {\varvec{e}}_{\theta }$, where $\theta$ is the polar angle measured from the squirmer orientation direction ${\varvec{e}}$, and ${\varvec{e}}_{\theta }$ is the unit vector tangent to the squirmer surface. This model introduces two model parameters: $B_{1}$ defines the self-propulsion strength and $\beta$ represents swimming mechanism. Depending on the swimming mechanism, we can have the following three types of swimmers: puller for $\beta > 0$ (e.g. *C. reinhardtii* cells), pusher for $\beta < 0$ (e.g. *E. coli*), and neutral for $\beta = 0$ (e.g. Volvox or Paramecium). It is assumed that the mass density of squirmer is same as the suspending fluid (i.e. the squirmer is neutrally buoyant). Thus, the mass of the squirmer is given by $M_{s} = \rho _{f}V_{s}$ (with squirmer volume $V_{s} = \frac{4}{3} \pi R^3$) and the moment of inertia of the squirmer is given by $I_{s} = \frac{2}{5} M_{s} R^2$. The squirmer has a center-of-mass position ${\varvec{R}}$, orientation ${\varvec{e}}$, translational velocity ${\varvec{V}}$ and angular velocity ${\varvec{\Omega }}$.

#### Virtual particles

In the simulation domain, $N_{f}$ fluid particles are distributed outside the squirmer body. Additionally, we use $N_{\text {vp}}$ pointlike virtual particles with the same mass as the fluid particles. The virtual particles are uniformly distributed throughout the volume of the squirmer with the same density as the fluid particles outside the squirmer (see Fig. 1). The purpose of the virtual particles is the implementation of the boundary conditions at the squirmer’s surface, and in the present particle-based framework the result is twofold [33, 39]: (i) precise implementation of the no-slip boundary condition and (ii) imposition of surface anchoring condition. In addition to position ${\varvec{r}}_{i}^{\text {vp}}$ and velocity ${\varvec{v}}_{i}^{\text {vp}}$, each virtual particle is also endowed with a tensor order parameter ${\varvec{q}}_{i}^{\text {vp}}$. A uniform distribution of virtual particles inside the squirmer is generated at each time step. The velocities are assigned as [38]

8 $$\begin{aligned} {\varvec{v}}_{i}^{\text {vp}} = {\varvec{V}} + {\varvec{\Omega }} \times ({\varvec{r}}_{i}^{\text {vp}} - {\varvec{R}}) + {\varvec{v}}_{s} + {\varvec{v}}_{i}^{\mathrm{{ran}}}, \end{aligned}$$

where the velocity of virtual particles consists of the rigid body motion of squirmer, surface velocity of squirmer, and a random velocity (with Cartesian components drawn from a Gaussian distribution of zero mean and standard deviation $\sqrt{k_{B}T_{0}/m_{0}}$). The extra degrees of freedom of the virtual particles, ${\varvec{q}}_{i}^{\text {vp}}$, allows us to implement the surface anchoring condition by simply prescribing $q_{\alpha \beta }^{\text {vp}} = S^{\text {vp}}(3n_{\alpha }^{\text {vp}}n_{\beta }^{\text {vp}} - \delta _{\alpha \beta })/2$, where $S^{\text {vp}}$ is the preferred nematic order on the squirmer’s surface and ${\varvec{n}}^{\text {vp}}$ is the preferred director orientation at the squirmer’s surface. This method effectively imposes infinitely strong anchoring condition on the squirmer’s surface. With these choices, the virtual particles take part in streaming and collision steps as discussed below.

#### Implementation of a moving squirmer

During the streaming step, the squirmer motion is updated by employing an MD-like step in which we integrate the equation of motion in the following form

9 $$\begin{aligned}&{\varvec{R}}(t+\Delta t) = {\varvec{R}}(t) + {\varvec{V}}(t) \Delta t + {\varvec{F}}(t) \frac{\Delta t^{2}}{2M_{s}}, \end{aligned}$$

10 $$\begin{aligned}&{\varvec{e}}(t+\Delta t) = {\varvec{e}}(t) + ({\varvec{\Omega }}(t) \times {\varvec{e}}(t)) \Delta t + ({\varvec{T}}(t) \times {\varvec{e}}(t)) \frac{\Delta t^{2}}{2I_{s}},\nonumber \\ \end{aligned}$$

11 $$\begin{aligned}&{\varvec{V}}(t+\Delta t) = {\varvec{V}}(t) + {\varvec{F}}(t) \frac{\Delta t}{M_{s}}, \end{aligned}$$

12 $$\begin{aligned}&{\varvec{\Omega }}(t+\Delta t) = {\varvec{\Omega }}(t) + {\varvec{T}}(t) \frac{\Delta t}{I_{s}}, \end{aligned}$$

where ${\varvec{F}}$ and ${\varvec{T}}$ are force and torque acting on the squirmer due to anisotropic viscous stress and elastic stress present in nematic LCs. We calculate the force and torque on the squirmer as ${\varvec{F}} = \sum _{j=1}^{N_\text {vp}} {\varvec{f}}_{j}^\text {vp}$ and ${\varvec{T}} = \sum _{j=1}^{N_\text {vp}} (({\varvec{r}}_{j}^{\text {vp}} - {\varvec{R}}) \times {\varvec{f}}_{j}^\text {vp})$. This approach by construction ensures the conservation of global momentum because the momentum lost by fluid particles is transferred to the squirmer via the coupling through virtual particles.

Note that the fluid particles interact with the squirmer via collision during the streaming step [46]. To impose the no-slip condition on the squirmer’s surface, we use the bounce-back rule (instead of using an interaction potential between squirmer and fluid particles [47]) whenever the fluid particles collide with the squirmer during the streaming step. Following a course-grained approach, it is assumed that fluid particle-squirmer collision happens at time $t + \Delta t /2$, which is valid provided the squirmer size is larger than the distance traversed by the fluid particle during streaming [48]. At the end of each streaming step, the change in momentum of fluid particles due to the fluid particle-squirmer collision is transferred back to the squirmer to balance the momentum.

**Fig. 2:**
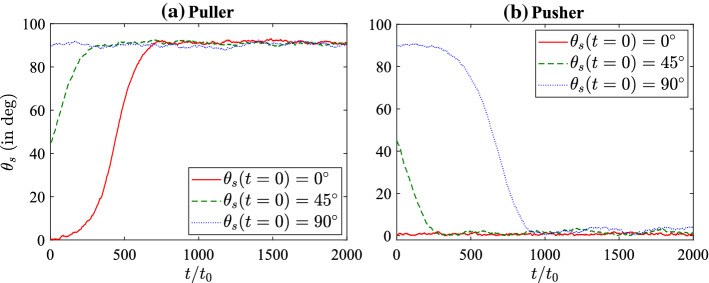
Variation of the orientation angle, $\theta _{s} = \cos ^{-1}({\varvec{e}} \cdot {\varvec{n}})$, with time for strong puller $(\beta = 5)$ and strong pusher $(\beta = -5)$ with $B_{1}=0.1$

During the collision step, the squirmer is coupled to the fluid via the virtual particles [46]. These virtual particles take part in the collision step and they suffer change in momentum similar to fluid particles. At the end of the collision step, the change in momentum of virtual particles is also transferred to the squirmer to balance the momentum. A step-by-step algorithm is presented in Appendix A.

### Model parameters

In the MPCD framework, the collision cell length $a_{0}$, the mass of MPCD particles $m_{0}$ and thermal energy of fluid $k_{B}T_{0}$ are taken as the scales of length, mass and energy, respectively. The scales for velocity, time and viscosity can be obtained as $v_{0} = \sqrt{k_{B}T_{0}/m_{0}}$, $t_{0} = a_{0}/v_{0}$, and $\eta _{0} = m_{0}/a_{0}t_{0}$, respectively. The collision time step $\Delta t$ determines the fluid viscosity and to have a liquidlike behavior $\Delta t$ should be smaller than $t_{0}$ (that is, large Schmidt numbers [49]). In the present nematic MPCD-MD framework, $\Delta t$ is also associated with the accuracy with which the streaming step of the fluid particles and the squirmer is performed. To have a proper time resolution, we take $\Delta t = 0.01 t_{0}$. The fluid density is given as $\rho _{f} = \langle N_{c} \rangle m_{0}/a_{0}^3$, where we choose the mean number density of fluid particles $\langle N_{c} \rangle = 30$. To have a proper resolution, the squirmer size should be such that it spans the size of a couple of collision cells. We have observed that a convenient choice of squirmer’s radius $R \geqslant 5 a_{0}$.

The nematic fluid is described by six viscosity coefficients ($\mu _{1}$, $\mu _{2}$, $\beta _{1}$, $\beta _{4}$ (isotropic part), $\beta _{5}$ and $\beta _{6}$), three phenomenological constants ($\alpha _{F}$, $\beta _{F}$ and $\gamma _{F}$), and one elastic constant (*L*). In the present study, we have taken 5CB as a model LC fluid [50] and fixed the dimensionless coefficients as $\mu _{1} = 132.782 \eta _{0}$, $\mu _{2} = -268.132 \eta _{0}$, $\beta _{1} = -20.533 \eta _{0}$, $\beta _{4} = 232.548 \eta _{0}$, $\beta _{5} = 202.364 \eta _{0}$, $\beta _{6} = -65.768 \eta _{0}$, $L = 132.782 k_{B}T_{0}/a_{0}$, $\alpha _{F} = -21.037 k_{B}T_{0}/a_{0}^{3}$, $\beta _{F} = 98.174 k_{B}T_{0}/a_{0}^{3}$ and $\gamma _{F} = 49.087 k_{B}T_{0}/a_{0}^{3}$ with the equilibrium scalar order parameter as $S_{eq} = 0.615$. These dimensionless properties are obtained by performing a mapping between MPCD scales and physical scales, details of which can be found elsewhere [39].

**Fig. 3:**
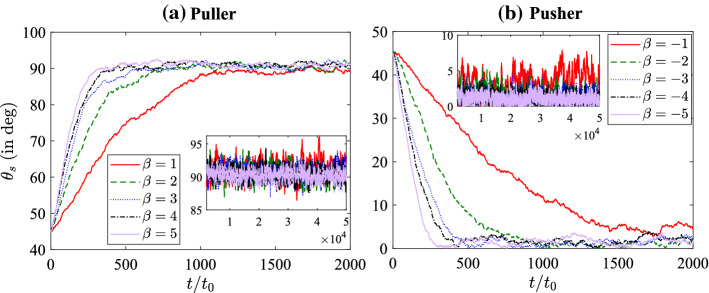
Variation of the orientation angle, $\theta _{s} = \cos ^{-1}({\varvec{e}} \cdot {\varvec{n}})$, with time for **a** pullers $(\beta > 0)$ and **b** pushers $(\beta < 0)$ with $B_{1}=0.1$

## Results

We apply the above-mentioned nematic MPCD-MD method to study the dynamics of a single squirmer in a bulk fluid with periodic boundary conditions in all dimensions. A squirmer of radius $R=6a_{0}$ with homeotropic surface anchoring is simulated in a three-dimensional nematic LC domain of size $60a_{0} \times 60a_{0} \times 60a_{0}$ for different values of $B_{1}$ and $\beta$.

**Fig. 4:**
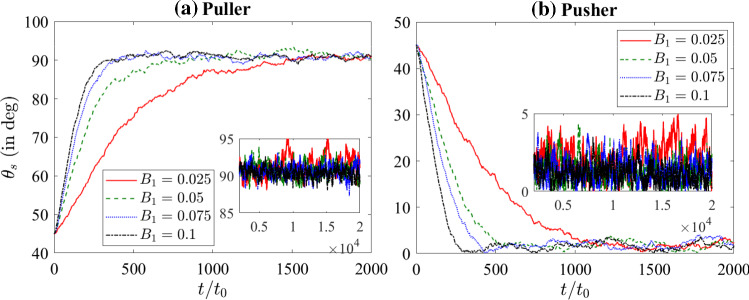
Variation of the orientation angle, $\theta _{s} = \cos ^{-1}({\varvec{e}} \cdot {\varvec{n}})$, with time for **a** puller $(\beta = 5)$ and **b** pusher $(\beta = -5)$ for different values of $B_{1}$

### Squirmer orientation

The temporal evolution of squirmer orientation can be represented by a single orientation angle $\theta _{s} = \cos ^{-1} ({\varvec{e}} \cdot {\varvec{n}})$, where ${\varvec{n}} = {\varvec{e}}_{z}$ is the nematic director in the bulk which is conventionally set along the *z*-axis far away from the squirmer (see Fig. 1 a). First, we investigate the temporal evolution of the orientation angle of a strong puller $(\beta = 5)$ and a strong pusher $(\beta = -5)$ in Fig. 2. Figure 2 a shows that the strong puller moves perpendicular to the nematic director at steady state (represented by $\theta _{s} = 90^{\circ }$ in the long time limit). Irrespective of the initial squirmer orientation $\theta _{s}(t=0)=0^{\circ }, 45^{\circ } \text { or } 90^{\circ }$, a strong puller always settles with an orientation perpendicular to the nematic director field. On the other hand, a strong pusher $(\beta = -5)$ shows a distinctly different orientation behavior as depicted in Fig. 2b. Irrespective of the initial squirmer orientation, a strong pusher always moves parallel to the nematic director at steady state (represented by $\theta _{s} = 0^{\circ }$ in the long time limit). Note that the tendency of pusher-type bacteria to align along the nematic director has been reported in several recent experiments [10, 14]. The squirmer orientation dynamics also compares well with the existing simulation and analytical results. Lintuvuori et al. [16] have shown recently that the squirmer encounters a nematodynamic toque due to the interaction between squirmer-generated flow and anisotropic viscosity. The approximate expression for the namatodynamic torque is of the form

13 $$\begin{aligned} {\varvec{T}}_{N} = - 8 \pi R^2 \eta \beta B_{1} ({\varvec{n}} \cdot {\varvec{e}})({\varvec{n}} \times {\varvec{e}}), \end{aligned}$$

where $\eta$ is an effective viscosity (negative for common nematic LCs). In the absence of thermal fluctuations, this nematodynamic torque governs the squirmer reorientation. A puller (pusher) moves perpendicular (parallel) to the nematic director.

Next, we investigate the effect of $\beta$ on the orientation dynamics of the squirmer. We carry out simulations with a fixed initial orientation of the squirmer, i.e. $\theta _{s}(t=0) = 45^{\circ }$ and calculate the squirmer’s orientation for pullers (Fig. 3a) and pushers (Fig. 3b) over a wide range of $\beta$. Figure 3 shows that with increasing $|\beta |$, the squirmer reaches its steady orientation more quickly and afterwards oscillates around the steady-state orientation; or in other words, the relaxation time to reach the steady-state orientation decreases with increasing magnitude of $\beta$. This behavior is in line with the existing lattice Boltzmann simulations [16] and can be explained by the fact that the magnitude of nematodynamic torque is proportional to the magnitude of $\beta$ (i.e. $|\beta |$) (refer to Eq. 13). The insets show the variation of $\theta _{s}$ in the long time. Note that the oscillations in orientation around the steady-state value is quite large (of the order of $5 ^{\circ }$) for small $|\beta |$. This is due to the fact that the nematodynamic toque is smaller in magnitude for small $|\beta |$ and thus thermal fluctuations affect the squirmer dynamics significantly. Note that in presence of thermal fluctuations the squirmer is acted upon by a stochastic torque which is generated due to collisions with the fluid particles. With increasing $|\beta |$, the oscillations reduce as the magnitude of nematodynamic torque dominates the thermal fluctuations.

**Fig. 5:**
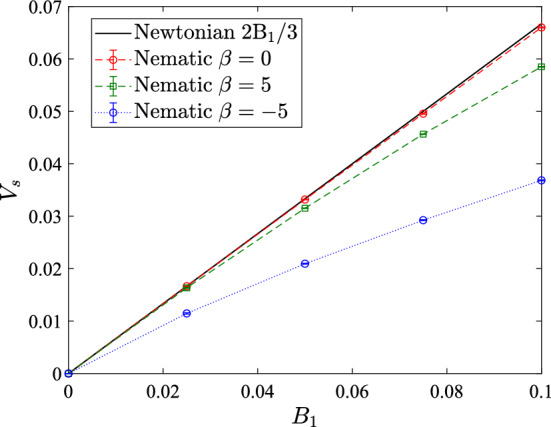
Variation of squirmer speed $(V_{s} = {\varvec{V}} \cdot {\varvec{e}})$ with $B_1$ for different values of $\beta$. Squirmer speed in Newtonian fluid, $V_{s} = 2B_{1}/3$, (analytical result) is also shown. Simulation results are shown by taking average over 5 independent runs for each parameter set

The effect of self-propulsion strength, $B_{1}$, on the squirmer orientation dynamics is presented in Fig. 4. Figure 4 shows that with increasing $B_{1}$, the squirmer quickly reaches its steady-state orientation and then oscillates around the steady-state orientation. The relaxation time reduces with increasing $B_{1}$. This can be explained by the fact that the magnitude of nematodynamic torque is proportional to $B_{1}$ (refer to Eq. 13). Thus, a faster squirmer will reorient to its steady orientation quickly as compared to a slower squirmer. When $B_{1}$ is small the nematodynamic toque is also small, and thermal fluctuations will strongly affect the squirmer’s dynamics. The insets show the variation of $\theta _{s}$ in the long time. Note that the oscillations in orientation around the steady-state value is quite large (of the order of $5 ^{\circ }$) for small $B_{1}$. This is due to the fact that the nematodynamic toque is smaller in magnitude for small $B_{1}$ and thus thermal fluctuations affect the squirmer dynamics significantly.

**Fig. 6:**
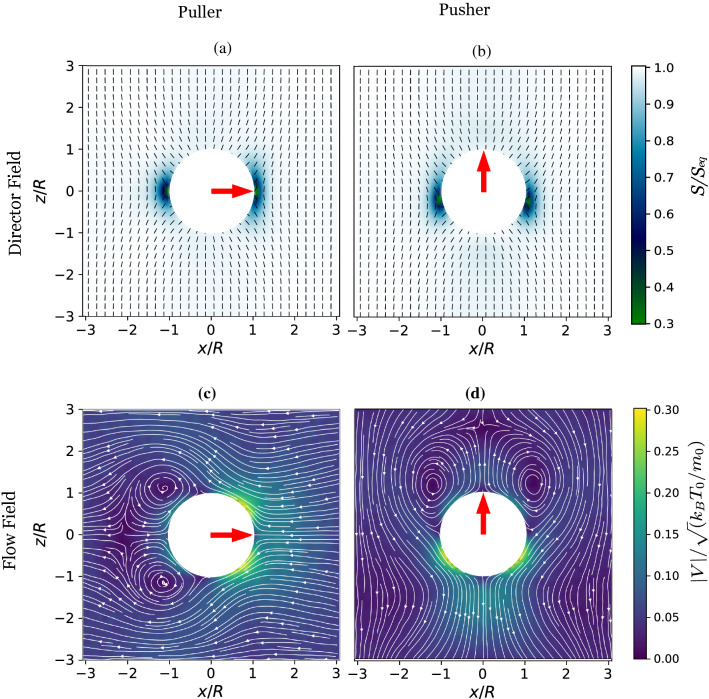
Time-averaged director field, ${\varvec{n}}$, (small black dashes) and scalar order parameter, $S/S_{eq}$, (color shading) around stationary **a** puller $(B_{1} = 0.1, \beta = 5)$, and **b** pusher $(B_{1} = 0.1, \beta = -5)$. Time-averaged streamlines (thin white lines with arrows) and velocity field (color shading) in the squirmer rest frame around stationary **c** puller $(B_{1} = 0.1, \beta = 5)$, and **d** pusher $(B_{1} = 0.1, \beta = -5)$. The white sphere represents the squirmer and the large red arrow represents the steady orientation of squirmer

### Squirmer speed

The speed of a squirmer, $V_{s} = {\varvec{V}} \cdot {\varvec{e}}$, with two surface modes $B_{1}$ and $\beta$ can be obtained analytically for the case of a Newtonian fluid as $V_{s} = 2B_{1}/3$. Notably, the squirmer speed is independent of $\beta$ and it increases linearly with $B_{1}$. Figure 5 shows the dependence of the squirmer’s speed on $B_{1}$ in nematic LC for puller $(\beta = 5)$, pusher $(\beta = -5)$, and neutral $(\beta = 0)$ squirmers. The squirmer velocities are measured in the steady orientation state. The predicted squirmer’s speed in the Newtonian fluid, $V_{s} = 2B_{1}/3$ is also plotted for the sake of comparison. Our simulation results show that the squirmer speed in nematic LC not only depends on $B_{1}$ but also depends on $\beta$. The swimming speed of pusher $(\beta = -5)$ is considerably smaller than in a Newtonian medium, and also smaller than the swimming speed of puller $(\beta = 5)$ and neutral $(\beta = 0)$ squirmer. Sokolov et al. [14] recently found experimentally that the average swimming speed of *B. subtilis* (pusher type bacterium) in lyotropic chromonic LC is about 2 times smaller than in a Newtonian medium. Figure 5 also shows that the variation of squirmer speed with $B_{1}$ is nonlinear for strong pusher. Note that the behaviour of a neutral squirmer is very similar in both Newtonian and nematic fluids.

### Nematic order and flow field around the squirmer

Figure 6a, b shows the time-averaged nematic director field $({\varvec{n}})$ and scalar order parameter $(S/S_{eq})$ for a puller and a pusher in stationary condition. On account of the homeotropic anchoring condition at the squirmer’s surface, a Saturn ring topological defect forms around the spherical squirmer. At steady state, the puller orients perpendicular to ${\varvec{n}}$, while the pusher orients parallel to ${\varvec{n}}$. Note that the structure of the Saturn ring remains very similar for both puller and pusher even for $B_{1}=0.1$. The only noticeable change is observed in the pusher for which the Saturn ring is slightly advected towards the rear end of the squirmer, downstream of the local flow. This fact is in agreement with experiments [51] and full MD simulations of nematic colloids in a flow [52]. Figure 6c, d show the time-averaged streamlines and velocity field. A closer look into these flow fields reveals that the streamlines for pusher are slightly more elongated along the *z* direction (parallel to the director); this is due to the fact the resistance to flow is smaller in the direction of the director. On the other hand, for the puller the streamlines are more compressed. Inspecting Fig. 6a, b can help rationalize the asymmetry in the speed of squirmers between pullers and pushers (see Fig. 5). Figure 6a shows that for pullers the Saturn ring topological defect induced by the squirmer on the nematic host is directly in front of the self-propulsion direction. The drastic reduction of the nematic order parameter at the leading edge of the squirmer reduces the local viscosity and thus allows it to move faster than the pusher.

## Conclusions

We have proposed a particle-based mesoscopic simulation method for microswimmers in nematic LCs. The existing nematic MPCD method is extended by combining it with an MD scheme for squirmer. We have tested this nematic MPCD-MD method for a single squirmer in an unbounded nematic LC medium. We correctly reproduce the orientation dynamics of squirmer in nematic LCs. The proposed method could be easily extended to study the collective dynamics of multiple squirmers in LCs.

We find that the speed of a squirmer moving within a nematic fluid depends nonlinearly on both $B_1$ (setting the self-propulsion strength) and $\beta$ (setting the type of swimmer.

## Nematic MPCD–MD algorithm

Below we provide a step–by–step algorithm of the nematic MPCD-MD method. These steps have been implemented on CUDA-capable GPU. At the beginning, the system is initialized by assigning the initial position, orientation, translational velocity and rotational velocity to the squirmer as ${\varvec{R}}(t=0) = {\varvec{0}}$ (i.e. at the middle of the simulation domain), ${\varvec{e}}(t=0) = \sin \theta _{s}(t=0) {\varvec{e}}_{x} + \cos \theta _{s}(t=0) {\varvec{e}}_{z}$ (with $\theta _{s}$ as the orientation of the squirmer measured from the *z*-axis), $\varvec{V}(t=0) = {\varvec{0}}$ and $\varvec{\Omega }(t=0) = {\varvec{0}}$. Next, the position, velocity and tensor order parameter of fluid and virtual particles are initialized. The fluid particles (outside the squirmer) and the virtual particles (inside the squirmer) are uniformly distributed. The number density of both fluid and virtual particles is $\langle N_{c} \rangle = 30$. The Cartesian velocity components of fluid and virtual particles are drawn from a Gaussian distribution with zero mean and standard deviation $\sqrt{k_{B}T_{0}/m_{0}}$. Additionally, the surface velocity of the squirmer ${\varvec{v}}_{s}$ is added into the velocity of virtual particles. The nematic orientational order is initialized by prescribing the particle-level tensor order parameter to fluid particles, while the homeotropic surface anchoring is imposed by prescribing the particle-level tensor order parameter to virtual particles.

1. The whole domain is divided into cubic cells of side length $a_{0}$. The fluid and virtual particles are sorted in these cells and cell-level quantities such as $N_{c}$, ${\varvec{V}}$, ${\varvec{Q}}$, ${\varvec{g}}$ and ${\varvec{f}}$ are calculated. To calculate ${\varvec{g}}$ and ${\varvec{f}}$, we need to take numerical differentiation of different cell-level quantities that we do using central difference scheme (details can be found in Ref. [39]).
2. The evolution equation of the tensor order parameter is employed to calculate ${\varvec{q}}_{i}$.
3. Perform streaming step (update position and velocity) of the fluid particles and the squirmer following respective equations of motion for the time $\Delta t$. Periodic boundary condition is imposed by checking whether the particles or squirmer moved out of the simulation domain.
4. After the streaming step, if $N_{\text {bb}}$ particles ended up inside the squirmer then the bounce-back rule is employed for those particles [48]. To resolve the collision, both the fluid particle and squirmer are moved back in time by $\Delta t /2$. The collision point on the squirmer surface is taken to be the projection of the particle position on the squirmer’s surface calculated as ${\varvec{r}}_{i} \leftarrow {\varvec{R}} + R ({\varvec{r}}_{i} - {\varvec{R}})/|({\varvec{r}}_{i} - {\varvec{R}})|$. Application of the bounce–back rule modifies the particle velocity as ${\varvec{v}}_{i} \leftarrow {\varvec{v}}_{i} - {\varvec{J}}_{i}/m_{0}$, where the momentum transfer ${\varvec{J}}_{i} = 2 m_{0} ({\varvec{v}}_{i} - {\varvec{V}} - {\varvec{\Omega }} \times ({\varvec{r}}_{i} - {\varvec{R}}) - {\varvec{v}}_{s})$ which takes into account the rigid body motion of squirmer and surface velocity of squirmer. After exchanging momentum, the fluid particles perform streaming for the rest $\Delta t / 2$ time with the modified velocity. The squirmer is also moved for the rest $\Delta t / 2$ time. To conserve the momentum, the momentum lost by $N_{\text {bb}}$ fluid particles are transferred to the squirmer by modifying its translational and angular velocities as ${\varvec{V}} \leftarrow {\varvec{V}} + \sum _{i=1}^{N_{\text {bb}}} {\varvec{J}}_{i} / M_{s}$, and ${\varvec{\Omega }} \leftarrow {\varvec{\Omega }} + \sum _{i=1}^{N_{\text {bb}}} ({\varvec{r}}_{i} - {\varvec{R}}) \times {\varvec{J}}_{i} / I_{s}$.
5. Position and velocity of virtual particles are re-generated as per the updated position and velocity of the squirmer.
6. Instead of shifting the cells, all the particles are moved as ${\varvec{r}}_{i} \leftarrow ({\varvec{r}}_{i} + {\varvec{s}})$ (where the components of ${\varvec{s}}$ are drawn from a uniform distribution within the interval $[-a_{0}/2, a_{0}/2]$) to reestablish the Galilean invariance. At the boundaries of the simulation domain, periodic boundary condition is applied.
7. The fluid and virtual particles are sorted in cells and cell–level quantities such as $N_{c}$, ${\varvec{r}}_{c}$, ${\varvec{V}}_{c}$ and ${\varvec{\Pi }}_{c}$ are calculated.
8. The collision step is performed to get the updated velocity of the fluid and virtual particles.
9. To conserve momentum, the momentum gained by the virtual particles is given to the squirmer [48]. Considering the velocity of virtual particles before and after collision step to be ${\varvec{v}}_{i}^{\text {vp}}$ and ${\varvec{v}}_{i}^{\text {vp}'}$, respectively, the momentum transfer can be calculated as ${\varvec{J}}_{i} = m_{0} ({\varvec{v}}_{i}^{\text {vp}'} - {\varvec{v}}_{i}^{\text {vp}})$. The momentum gained by $N_{\text {vp}}$ virtual particles is transferred to the squirmer by modifying its translational and rotational velocities as ${\varvec{V}} \leftarrow {\varvec{V}} + \sum _{i=1}^{N_{\text {vp}}} {\varvec{J}}_{i} / M_{s}$, and ${\varvec{\Omega }} \leftarrow {\varvec{\Omega }} + \sum _{i=1}^{N_{\text {vp}}} ({\varvec{r}}_{i} - {\varvec{R}}) \times {\varvec{J}}_{i} / I_{s}$.
10. The particle–shift operation is performed in reverse, ${\varvec{r}}_{i} \leftarrow ({\varvec{r}}_{i} - {\varvec{s}})$, to shift back the fluid and virtual particles to their original position. At the boundaries of the simulation domain, periodic boundary condition is applied.
